# (2*E*)-2-[(2-Hy­droxy-4-meth­oxy­phen­yl)(phen­yl)methyl­idene]-*N*-phenyl­hydrazinecarboxamide dimethyl­formamide monosolvate

**DOI:** 10.1107/S1600536812017382

**Published:** 2012-04-25

**Authors:** C. F. Annie, Jinsa Mary Jacob, M. Sithambaresan, M. R. Prathapachandra Kurup

**Affiliations:** a Department of Applied Chemistry, Cochin University of Science and Technology, Kochi 682 022, India; b Department of Chemistry, Faculty of Science, Eastern University, Sri Lanka, Chenkalady, Sri Lanka

## Abstract

The title compound, C_21_H_19_N_3_O_3_·C_3_H_7_NO, adopts an *E* conformation with respect to the azomethine bond and crystallizes in the amide form. The dihedral angle between the rings lined to the C=N bond is 88.60 (12)°. The dimethyl­formamide solvent mol­ecule is disordered over two orientations with site occupancies of 0.684 (3) and 0.316 (3). The two N atoms of the hydrazinecarboxamide group are involved in inter­molecular N—H⋯O hydrogen bonds in which the dimethyl­formamide O atom acts as acceptor. The structure also features π–π inter­actions, with a centroid–centroid distance of 3.6561 (13) Å. Classical and non-classical intra­molecular O—H⋯N and C—H⋯O hydrogen bonds are also present.

## Related literature

For applications of hydrazinecarboxamide and its derivatives, see: Afrasiabi *et al.* (2005 ▶); Alam *et al.* (2010 ▶). For related structures, see: Siji *et al.* (2010 ▶); Reena & Kurup (2010 ▶); Sithambaresan & Kurup (2011 ▶). For standard bond-length data, see: March (1992 ▶); Kala *et al.* (2007 ▶). For the synthesis, see: Sreekanth *et al.* (2004 ▶).
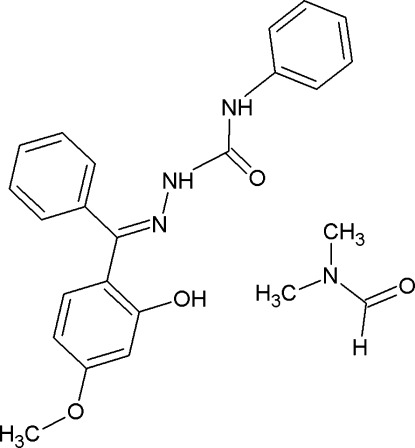


## Experimental

### 

#### Crystal data

C_21_H_19_N_3_O_3_·C_3_H_7_NO
*M* *_r_* = 434.49
Monoclinic, 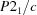

*a* = 13.1155 (7) Å
*b* = 16.9619 (11) Å
*c* = 10.7399 (5) Å
β = 105.509 (3)°
*V* = 2302.2 (2) Å^3^
*Z* = 4
Mo *K*α radiation
μ = 0.09 mm^−1^
*T* = 296 K
0.35 × 0.30 × 0.25 mm

#### Data collection

Bruker Kappa APEXII CCD diffractometer
Absorption correction: multi-scan (*SADABS*; Bruker, 2004 ▶) *T*_min_ = 0.970, *T*_max_ = 0.978
17400 measured reflections
4061 independent reflections
2875 reflections with *I* > 2σ(*I*)
*R*_int_ = 0.069

#### Refinement

*R*[*F* ^2^ > 2σ(*F* ^2^)] = 0.052
*wR*(*F* ^2^) = 0.158
*S* = 1.02
4061 reflections
311 parameters
10 restraints
H-atom parameters constrained
Δρ_max_ = 0.18 e Å^−3^
Δρ_min_ = −0.19 e Å^−3^

### 

Data collection: *APEX2* (Bruker, 2004 ▶); cell refinement: *APEX2* and *SAINT* (Bruker, 2004 ▶); data reduction: *SAINT* and *XPREP* (Bruker, 2004 ▶); program(s) used to solve structure: *SHELXS97* (Sheldrick, 2008 ▶); program(s) used to refine structure: *SHELXL97* (Sheldrick, 2008 ▶); molecular graphics: *ORTEP-3* (Farrugia, 1997 ▶) and *DIAMOND* (Brandenburg, 2010 ▶); software used to prepare material for publication: *SHELXL97* and *publCIF* (Westrip, 2010 ▶).

## Supplementary Material

Crystal structure: contains datablock(s) I, global. DOI: 10.1107/S1600536812017382/bv2201sup1.cif

Structure factors: contains datablock(s) I. DOI: 10.1107/S1600536812017382/bv2201Isup2.hkl

Supplementary material file. DOI: 10.1107/S1600536812017382/bv2201Isup3.cml

Additional supplementary materials:  crystallographic information; 3D view; checkCIF report

## Figures and Tables

**Table 1:** Hydrogen-bond geometry (Å, °)

*D*—H⋯*A*	*D*—H	H⋯*A*	*D*⋯*A*	*D*—H⋯*A*
O1—H1⋯N1	0.89	1.78	2.5655 (18)	147
N2—H2′⋯O4*A*	0.87	2.19	2.941 (12)	145
N2—H2′⋯O4*B*	0.87	2.22	2.96 (3)	143
N3—H3′⋯O4*A*	0.85	2.00	2.830 (7)	165
N3—H3′⋯O4*B*	0.85	2.07	2.882 (18)	161
C24*A*—H24*C*⋯O3^i^	0.96	2.62	3.400 (4)	139
C23*B*—H23*E*⋯O3^i^	0.96	2.31	3.157 (13)	147
C23*B*—H23*F*⋯O1^i^	0.96	2.59	3.473 (13)	153

Symmetry code: (i) 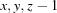.

**Fig. 1.**
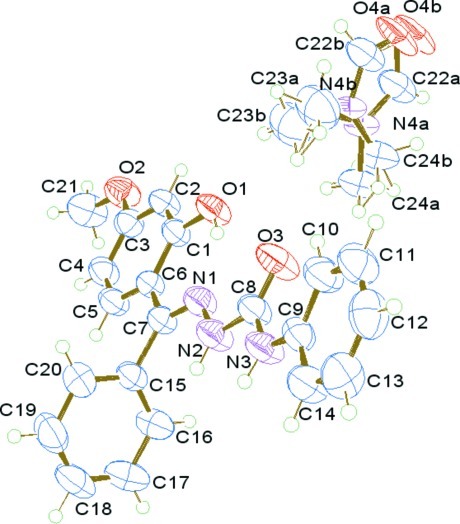
The molecular structure of the title compound, showing the disordered DMF solvent molecule.

**Fig. 2.**
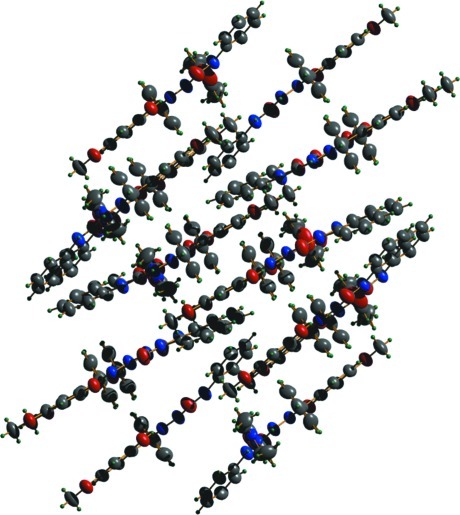
A view of the unit cell along c axis.

## supplementary crystallographic information

### Comment

Semicarbazones are compounds with versatile structural features (Siji *et al.*, 2010) and exhibit a wide range of bioactivities including anticancer (Afrasiabi *et al.*, 2005) and antiviral properties. Some of them are found to exhibit anticonvulsant activity with less neurotoxicity (Alam *et al.*, 2010).

The compound crystallizes in monoclinic *P*2_1_/*c* space group. The molecule exists in the *E* configuration with respect to C7=N1 bond (Sithambaresan and Kurup, 2011) which is confirmed by the torsion angle of -179.56 (15)° of N2—N1—C7—C6 moiety (Fig. 1). The torsion angle value of -3.9 (3)° corresponding to N1—N2—C8—O3 moiety supports the *cis* configuration of the O3 atom with respect to the nitrogen atom N1. Also the torsion angles of -1.6 (3)° and -1.2 (3)° for O1—C1—C6—C7 and C1—C6—C7—N1 moieties respectively confirm the *cis* configuration of phenolic oxygen O1 and azomethine nitrogen N1 and it favors intramolecular hydrogen bonding between N1 and H attached to O1.

The C7–N1 bond distance [1.290 (2) Å] is in conformity with a formal C=N bond [1.28 Å] (March, 1992) confirming the azomethine bond formation. The existence of semicarbazone in the amido form in the solid state is confirmed by the observed bond length of 1.210 (2) Å for C8–O3 bond which is identical to a formal C=O bond length [1.21 Å] (Kala *et al.*, 2007). The N1–N2 and N2–C8 bond distances of 1.364 (2) Å and 1.377 (2) Å respectively are greater than that for a double bond and less than that for a single bond which indicate appreciable delocalization of π-electron density over the semicarbazone chain (Reena & Kurup, 2010). Rings *Cg*1^ii^ (comprising atoms C1—C6) and *Cg*2^iii^ (comprising atoms C9—C14) make a dihedral angle of 14.43 (12)° with each other while rings *Cg*1^ii^ and *Cg*3^iv^ (comprising atoms C15—C20) are twisted away from each other by a dihedral angle of 88.60 (12)° [symmetry codes: (ii)1 - *x*,1 - *y*,1 - *z*; (iii)1 + *x*,3/2 - *y*,1/2 + *z*; (iv)*x*,3/2 - *y*,1/2 + *z*]. The dimethylformamide solvent molecule is disordered with site occupancies of 0.684 (3) and 0.316 (3).

Fig. 2 shows the packing diagram of the title compound along *c* axis. The nitrogen atoms, N2 and N3 of the hydrazine carboxamide moiety form classical intermolecular hydrogen bonds N2–H2'···O4^i^ and N3–H3'···O4^i^ respectively in which oxygen atom of the solvent dimethylformamide functions as acceptor and two non classical intramolecular hydrogen bonding interactions also found in the molecule and the DMFsolvent (Table 1). A prominent π···π interaction is observed with a centroid-centroid distance of 3.6561 (13) Å between the *Cg*1^ii^ rings of two molecules. Other short ring interactions are weak as they correspond to a distance greater than 4 Å. Two types of C–H···π interactions with H···π distances of 3.3114 and 3.3197 Å are also present in the crystal system.

### Experimental

The title compound was prepared by adapting a reported procedure (Sreekanth *et al.*, 2004). To a warm methanolic solution of *N*-phenylsemicarbazide (0.302 g, 2 mmol), a methanolic solution of 2-hydroxy-4-methoxybenzophenone (0.456 g, 2 mmol) was added and the resulting solution was refluxed for 2 h after adding a drop of conc. HCl. On cooling the solution colorless crystals were separated out. Single crystals suitable for X-ray diffraction studies were obtained by slow evaporation of its solution in a 1:1 mixture of methanol and DMF.

### Refinement

All H atoms on C were placed in calculated positions, guided by difference maps, with C–H bond distances 0.93–0.97 Å. H atoms were assigned as *U*_iso_=1.2Ueq (1.5 for Me). N2–H2', N3–H3' and O1–H1 H atoms were located from difference maps and restrained using *DFIX* instructions. All the C, N and O atoms of the dimethylformamide molecule are disordered over two sites A and B with relative occupancies of 0.637 (4) and 0.363 (4) respectively..

### Crystal data

**Table:**

C_21_H_19_N_3_O_3_·C_3_H_7_NO	*F*(000) = 920
*M**_r_* = 434.49	*D*_x_ = 1.254 Mg m^−^^3^
Monoclinic, *P*2_1_/*c*	Mo *K*α radiation, λ = 0.71073 Å
Hall symbol: -P 2ybc	Cell parameters from 5896 reflections
*a* = 13.1155 (7) Å	θ = 2.4–28.2°
*b* = 16.9619 (11) Å	µ = 0.09 mm^−^^1^
*c* = 10.7399 (5) Å	*T* = 296 K
β = 105.509 (3)°	Block, colourless
*V* = 2302.2 (2) Å^3^	0.35 × 0.30 × 0.25 mm
*Z* = 4	

### Data collection

**Table:**

Bruker Kappa APEXII CCD diffractometer	4061 independent reflections
Radiation source: fine-focus sealed tube	2875 reflections with *I* > 2σ(*I*)
Graphite monochromator	*R*_int_ = 0.069
ω and φ scan	θ_max_ = 25.0°, θ_min_ = 2.3°
Absorption correction: multi-scan (*SADABS*; Bruker, 2004)	*h* = −15→15
*T*_min_ = 0.970, *T*_max_ = 0.978	*k* = −19→20
17400 measured reflections	*l* = −12→12

### Refinement

**Table:**

Refinement on *F*^2^	Primary atom site location: structure-invariant direct methods
Least-squares matrix: full	Secondary atom site location: difference Fourier map
*R*[*F*^2^ > 2σ(*F*^2^)] = 0.052	Hydrogen site location: inferred from neighbouring sites
*wR*(*F*^2^) = 0.158	H-atom parameters constrained
*S* = 1.02	*w* = 1/[σ^2^(*F*_o_^2^) + (0.0824*P*)^2^ + 0.2581*P*] where *P* = (*F*_o_^2^ + 2*F*_c_^2^)/3
4061 reflections	(Δ/σ)_max_ < 0.001
311 parameters	Δρ_max_ = 0.18 e Å^−^^3^
10 restraints	Δρ_min_ = −0.19 e Å^−^^3^

### Special details

**Table:**

Geometry. All esds (except the esd in the dihedral angle between two l.s. planes) are estimated using the full covariance matrix. The cell esds are taken into account individually in the estimation of esds in distances, angles and torsion angles; correlations between esds in cell parameters are only used when they are defined by crystal symmetry. An approximate (isotropic) treatment of cell esds is used for estimating esds involving l.s. planes.
Refinement. Refinement of F^2^ against ALL reflections. The weighted R-factor wR and goodness of fit S are based on F^2^, conventional R-factors R are based on F, with F set to zero for negative F^2^. The threshold expression of F^2^ > 2sigma(F^2^) is used only for calculating R-factors(gt) etc. and is not relevant to the choice of reflections for refinement. R-factors based on F^2^ are statistically about twice as large as those based on F, and R- factors based on ALL data will be even larger.

### Fractional atomic coordinates and isotropic or equivalent isotropic displacement parameters (Å^2^)

**Table:**

	*x*	*y*	*z*	*U*_iso_*/*U*_eq_	Occ. (<1)
O1	0.84998 (10)	0.61778 (8)	1.04160 (12)	0.0666 (4)	
H1	0.7974	0.6191	0.9700	0.100*	
O2	1.21655 (11)	0.59166 (9)	1.18461 (13)	0.0740 (4)	
O3	0.57307 (11)	0.63119 (10)	0.84928 (12)	0.0826 (5)	
N1	0.75944 (12)	0.61397 (9)	0.79832 (14)	0.0588 (4)	
N2	0.66741 (12)	0.61639 (10)	0.70205 (14)	0.0655 (5)	
H2'	0.6700	0.6187	0.6222	0.079*	
N3	0.48988 (12)	0.63420 (11)	0.63350 (14)	0.0663 (5)	
H3'	0.5040	0.6304	0.5610	0.080*	
C1	0.94202 (14)	0.60977 (10)	1.00767 (16)	0.0520 (4)	
C2	1.03416 (15)	0.60499 (11)	1.10594 (16)	0.0581 (5)	
H2	1.0311	0.6074	1.1914	0.070*	
C3	1.13084 (14)	0.59664 (11)	1.07944 (17)	0.0559 (4)	
C4	1.13607 (15)	0.59357 (12)	0.95264 (17)	0.0609 (5)	
H4	1.2009	0.5881	0.9338	0.073*	
C5	1.04372 (14)	0.59881 (11)	0.85454 (17)	0.0575 (5)	
H5	1.0479	0.5971	0.7695	0.069*	
C6	0.94474 (13)	0.60652 (10)	0.87711 (15)	0.0496 (4)	
C7	0.84867 (14)	0.60951 (10)	0.76958 (16)	0.0509 (4)	
C8	0.57499 (15)	0.62767 (12)	0.73737 (17)	0.0590 (5)	
C9	0.38600 (14)	0.65561 (11)	0.63201 (16)	0.0561 (5)	
C10	0.35631 (16)	0.68054 (13)	0.73941 (19)	0.0705 (6)	
H10	0.4059	0.6834	0.8194	0.085*	
C11	0.25230 (18)	0.70127 (14)	0.7270 (2)	0.0804 (6)	
H11	0.2324	0.7176	0.7998	0.097*	
C12	0.17775 (17)	0.69846 (14)	0.6102 (2)	0.0794 (6)	
H12	0.1080	0.7128	0.6036	0.095*	
C13	0.20729 (17)	0.67438 (15)	0.5040 (2)	0.0811 (6)	
H13	0.1576	0.6724	0.4240	0.097*	
C14	0.31036 (16)	0.65306 (14)	0.51496 (19)	0.0722 (6)	
H14	0.3295	0.6365	0.4418	0.087*	
C15	0.85612 (14)	0.60722 (11)	0.63283 (16)	0.0527 (4)	
C16	0.85331 (16)	0.53656 (13)	0.57021 (17)	0.0710 (6)	
H16	0.8457	0.4899	0.6124	0.085*	
C17	0.86165 (18)	0.53419 (16)	0.4453 (2)	0.0847 (7)	
H17	0.8602	0.4861	0.4033	0.102*	
C18	0.87206 (17)	0.60280 (18)	0.3829 (2)	0.0831 (7)	
H18	0.8780	0.6014	0.2986	0.100*	
C19	0.87374 (19)	0.67305 (17)	0.4443 (2)	0.0896 (7)	
H19	0.8803	0.7196	0.4012	0.108*	
C20	0.86589 (17)	0.67617 (14)	0.56947 (19)	0.0748 (6)	
H20	0.8672	0.7245	0.6109	0.090*	
C21	1.31791 (17)	0.58519 (18)	1.1620 (2)	0.0898 (7)	
H21A	1.3203	0.5388	1.1116	0.135*	
H21B	1.3708	0.5815	1.2431	0.135*	
H21C	1.3312	0.6309	1.1160	0.135*	
O4A	0.5772 (14)	0.6210 (6)	0.4203 (10)	0.100 (2)	0.684 (3)
N4A	0.6036 (4)	0.59304 (18)	0.2263 (4)	0.0615 (10)	0.684 (3)
C22A	0.5958 (2)	0.5725 (2)	0.3396 (3)	0.0780 (9)	0.684 (3)
H22A	0.6043	0.5196	0.3627	0.094*	0.684 (3)
C23A	0.6011 (5)	0.6733 (3)	0.1866 (5)	0.1115 (15)	0.684 (3)
H23A	0.5797	0.7059	0.2481	0.167*	0.684 (3)
H23B	0.5517	0.6791	0.1032	0.167*	0.684 (3)
H23C	0.6703	0.6888	0.1818	0.167*	0.684 (3)
C24A	0.6284 (3)	0.5357 (2)	0.1380 (4)	0.0957 (11)	0.684 (3)
H24A	0.6188	0.4834	0.1672	0.144*	0.684 (3)
H24B	0.7005	0.5423	0.1356	0.144*	0.684 (3)
H24C	0.5822	0.5435	0.0530	0.144*	0.684 (3)
O4B	0.576 (3)	0.5968 (15)	0.421 (2)	0.100 (2)	0.316 (3)
N4B	0.6081 (12)	0.6247 (6)	0.2322 (10)	0.0615 (10)	0.316 (3)
C22B	0.5949 (5)	0.6475 (5)	0.3420 (6)	0.0780 (9)	0.316 (3)
H22B	0.5990	0.7008	0.3633	0.094*	0.316 (3)
C23B	0.6333 (13)	0.6804 (8)	0.1434 (12)	0.1115 (15)	0.316 (3)
H23D	0.6241	0.7331	0.1712	0.167*	0.316 (3)
H23E	0.5872	0.6720	0.0586	0.167*	0.316 (3)
H23F	0.7054	0.6731	0.1413	0.167*	0.316 (3)
C24B	0.5982 (9)	0.5433 (6)	0.1957 (9)	0.0957 (11)	0.316 (3)
H24D	0.5607	0.5157	0.2478	0.144*	0.316 (3)
H24E	0.6673	0.5207	0.2085	0.144*	0.316 (3)
H24F	0.5599	0.5390	0.1062	0.144*	0.316 (3)

### Atomic displacement parameters (Å^2^)

**Table:**

	*U*^11^	*U*^22^	*U*^33^	*U*^12^	*U*^13^	*U*^23^
O1	0.0586 (8)	0.0987 (11)	0.0499 (7)	0.0052 (6)	0.0275 (6)	−0.0003 (6)
O2	0.0609 (8)	0.1036 (11)	0.0558 (8)	0.0070 (7)	0.0124 (6)	0.0008 (7)
O3	0.0688 (9)	0.1380 (14)	0.0465 (8)	0.0131 (8)	0.0250 (6)	0.0061 (7)
N1	0.0527 (9)	0.0780 (11)	0.0483 (8)	0.0002 (7)	0.0181 (7)	−0.0024 (7)
N2	0.0509 (9)	0.1028 (13)	0.0464 (8)	0.0032 (8)	0.0191 (7)	−0.0020 (7)
N3	0.0544 (9)	0.1032 (13)	0.0464 (8)	0.0042 (8)	0.0224 (7)	−0.0056 (8)
C1	0.0570 (10)	0.0564 (11)	0.0489 (9)	−0.0002 (8)	0.0249 (8)	−0.0001 (7)
C2	0.0648 (12)	0.0707 (12)	0.0426 (9)	0.0019 (9)	0.0208 (8)	0.0010 (8)
C3	0.0565 (11)	0.0599 (11)	0.0506 (10)	0.0009 (8)	0.0132 (8)	0.0013 (8)
C4	0.0533 (11)	0.0763 (13)	0.0577 (11)	0.0004 (9)	0.0230 (9)	−0.0020 (9)
C5	0.0578 (11)	0.0731 (13)	0.0466 (9)	−0.0014 (9)	0.0224 (8)	−0.0018 (8)
C6	0.0557 (10)	0.0525 (10)	0.0446 (9)	−0.0014 (8)	0.0204 (8)	−0.0013 (7)
C7	0.0552 (10)	0.0554 (10)	0.0468 (9)	0.0004 (8)	0.0216 (8)	−0.0019 (7)
C8	0.0562 (11)	0.0774 (13)	0.0483 (10)	0.0011 (9)	0.0224 (8)	0.0005 (8)
C9	0.0541 (10)	0.0655 (12)	0.0545 (10)	−0.0016 (8)	0.0245 (8)	−0.0001 (8)
C10	0.0660 (12)	0.0894 (15)	0.0636 (12)	0.0022 (10)	0.0301 (10)	−0.0071 (10)
C11	0.0787 (15)	0.0872 (16)	0.0902 (15)	0.0045 (12)	0.0481 (13)	−0.0083 (12)
C12	0.0601 (13)	0.0776 (15)	0.1070 (18)	0.0068 (11)	0.0335 (13)	0.0089 (12)
C13	0.0610 (13)	0.0981 (18)	0.0811 (14)	0.0040 (11)	0.0138 (11)	0.0091 (12)
C14	0.0657 (13)	0.0950 (16)	0.0576 (11)	0.0047 (11)	0.0197 (10)	0.0009 (10)
C15	0.0483 (9)	0.0676 (12)	0.0453 (9)	0.0019 (8)	0.0180 (7)	−0.0003 (8)
C16	0.0888 (15)	0.0769 (14)	0.0508 (10)	0.0136 (11)	0.0248 (10)	0.0000 (9)
C17	0.0972 (17)	0.1058 (19)	0.0559 (12)	0.0201 (14)	0.0286 (11)	−0.0127 (12)
C18	0.0656 (13)	0.142 (2)	0.0486 (11)	−0.0003 (13)	0.0267 (10)	0.0035 (13)
C19	0.0954 (17)	0.113 (2)	0.0636 (13)	−0.0207 (14)	0.0274 (12)	0.0217 (13)
C20	0.0877 (15)	0.0799 (15)	0.0613 (12)	−0.0139 (11)	0.0276 (10)	0.0036 (10)
C21	0.0567 (13)	0.134 (2)	0.0743 (14)	0.0100 (13)	0.0106 (10)	−0.0093 (13)
O4A	0.0932 (13)	0.156 (7)	0.0592 (9)	0.019 (6)	0.0347 (9)	−0.010 (3)
N4A	0.0719 (13)	0.064 (3)	0.0525 (11)	−0.004 (3)	0.0242 (10)	−0.004 (2)
C22A	0.0719 (17)	0.115 (3)	0.0520 (14)	0.0056 (19)	0.0260 (13)	0.0029 (17)
C23A	0.146 (5)	0.099 (3)	0.099 (4)	0.018 (3)	0.048 (3)	0.018 (3)
C24A	0.122 (3)	0.103 (3)	0.073 (2)	−0.008 (2)	0.045 (2)	−0.022 (2)
O4B	0.0932 (13)	0.156 (7)	0.0592 (9)	0.019 (6)	0.0347 (9)	−0.010 (3)
N4B	0.0719 (13)	0.064 (3)	0.0525 (11)	−0.004 (3)	0.0242 (10)	−0.004 (2)
C22B	0.0719 (17)	0.115 (3)	0.0520 (14)	0.0056 (19)	0.0260 (13)	0.0029 (17)
C23B	0.146 (5)	0.099 (3)	0.099 (4)	0.018 (3)	0.048 (3)	0.018 (3)
C24B	0.122 (3)	0.103 (3)	0.073 (2)	−0.008 (2)	0.045 (2)	−0.022 (2)

### Geometric parameters (Å, º)

**Table:**

O1—C1	1.3579 (19)	C15—C20	1.376 (3)
O1—H1	0.8851	C16—C17	1.376 (3)
O2—C3	1.367 (2)	C16—H16	0.9300
O2—C21	1.418 (2)	C17—C18	1.368 (3)
O3—C8	1.210 (2)	C17—H17	0.9300
N1—C7	1.290 (2)	C18—C19	1.359 (4)
N1—N2	1.364 (2)	C18—H18	0.9300
N2—C8	1.377 (2)	C19—C20	1.376 (3)
N2—H2'	0.8683	C19—H19	0.9300
N3—C8	1.355 (2)	C20—H20	0.9300
N3—C9	1.406 (2)	C21—H21A	0.9600
N3—H3'	0.8495	C21—H21B	0.9600
C1—C2	1.378 (3)	C21—H21C	0.9600
C1—C6	1.413 (2)	O4A—C22A	1.265 (10)
C2—C3	1.379 (2)	N4A—C22A	1.295 (4)
C2—H2	0.9300	N4A—C23A	1.424 (6)
C3—C4	1.383 (2)	N4A—C24A	1.455 (5)
C4—C5	1.379 (3)	C22A—H22A	0.9300
C4—H4	0.9300	C23A—H23A	0.9600
C5—C6	1.389 (2)	C23A—H23B	0.9600
C5—H5	0.9300	C23A—H23C	0.9600
C6—C7	1.465 (2)	C24A—H24A	0.9600
C7—C15	1.498 (2)	C24A—H24B	0.9600
C9—C14	1.378 (3)	C24A—H24C	0.9600
C9—C10	1.379 (2)	O4B—C22B	1.274 (11)
C10—C11	1.380 (3)	N4B—C22B	1.295 (10)
C10—H10	0.9300	N4B—C24B	1.432 (10)
C11—C12	1.369 (3)	N4B—C23B	1.442 (11)
C11—H11	0.9300	C22B—H22B	0.9300
C12—C13	1.362 (3)	C23B—H23D	0.9600
C12—H12	0.9300	C23B—H23E	0.9600
C13—C14	1.374 (3)	C23B—H23F	0.9600
C13—H13	0.9300	C24B—H24D	0.9600
C14—H14	0.9300	C24B—H24E	0.9600
C15—C16	1.370 (3)	C24B—H24F	0.9600
			
C1—O1—H1	108.1	C9—C14—H14	119.3
C3—O2—C21	117.70 (15)	C16—C15—C20	119.64 (17)
C7—N1—N2	119.74 (14)	C16—C15—C7	120.25 (16)
N1—N2—C8	117.46 (14)	C20—C15—C7	120.11 (17)
N1—N2—H2'	119.3	C15—C16—C17	120.4 (2)
C8—N2—H2'	122.4	C15—C16—H16	119.8
C8—N3—C9	127.79 (14)	C17—C16—H16	119.8
C8—N3—H3'	114.5	C18—C17—C16	119.8 (2)
C9—N3—H3'	117.2	C18—C17—H17	120.1
O1—C1—C2	117.44 (14)	C16—C17—H17	120.1
O1—C1—C6	122.02 (16)	C19—C18—C17	119.97 (19)
C2—C1—C6	120.54 (15)	C19—C18—H18	120.0
C1—C2—C3	120.95 (15)	C17—C18—H18	120.0
C1—C2—H2	119.5	C18—C19—C20	120.8 (2)
C3—C2—H2	119.5	C18—C19—H19	119.6
O2—C3—C2	115.74 (15)	C20—C19—H19	119.6
O2—C3—C4	124.42 (16)	C15—C20—C19	119.4 (2)
C2—C3—C4	119.85 (17)	C15—C20—H20	120.3
C5—C4—C3	119.02 (16)	C19—C20—H20	120.3
C5—C4—H4	120.5	O2—C21—H21A	109.5
C3—C4—H4	120.5	O2—C21—H21B	109.5
C4—C5—C6	122.93 (15)	H21A—C21—H21B	109.5
C4—C5—H5	118.5	O2—C21—H21C	109.5
C6—C5—H5	118.5	H21A—C21—H21C	109.5
C5—C6—C1	116.71 (16)	H21B—C21—H21C	109.5
C5—C6—C7	120.84 (14)	C22A—N4A—C23A	122.5 (4)
C1—C6—C7	122.44 (15)	C22A—N4A—C24A	121.3 (3)
N1—C7—C6	117.22 (14)	C23A—N4A—C24A	115.8 (4)
N1—C7—C15	122.50 (15)	O4A—C22A—N4A	123.1 (5)
C6—C7—C15	120.28 (14)	O4A—C22A—H22A	118.4
O3—C8—N3	125.60 (16)	N4A—C22A—H22A	118.4
O3—C8—N2	122.28 (17)	C22B—N4B—C24B	120.9 (10)
N3—C8—N2	112.12 (14)	C22B—N4B—C23B	121.1 (9)
C14—C9—C10	118.51 (17)	C24B—N4B—C23B	118.0 (9)
C14—C9—N3	117.47 (15)	O4B—C22B—N4B	119.8 (14)
C10—C9—N3	124.01 (17)	O4B—C22B—H22B	120.1
C9—C10—C11	119.4 (2)	N4B—C22B—H22B	120.1
C9—C10—H10	120.3	N4B—C23B—H23D	109.5
C11—C10—H10	120.3	N4B—C23B—H23E	109.5
C12—C11—C10	121.66 (19)	H23D—C23B—H23E	109.5
C12—C11—H11	119.2	N4B—C23B—H23F	109.5
C10—C11—H11	119.2	H23D—C23B—H23F	109.5
C13—C12—C11	118.96 (19)	H23E—C23B—H23F	109.5
C13—C12—H12	120.5	N4B—C24B—H24D	109.5
C11—C12—H12	120.5	N4B—C24B—H24E	109.5
C12—C13—C14	120.1 (2)	H24D—C24B—H24E	109.5
C12—C13—H13	120.0	N4B—C24B—H24F	109.5
C14—C13—H13	120.0	H24D—C24B—H24F	109.5
C13—C14—C9	121.45 (18)	H24E—C24B—H24F	109.5
C13—C14—H14	119.3		
			
C7—N1—N2—C8	−174.78 (16)	C8—N3—C9—C14	−175.6 (2)
O1—C1—C2—C3	179.87 (16)	C8—N3—C9—C10	5.9 (3)
C6—C1—C2—C3	−0.3 (3)	C14—C9—C10—C11	0.7 (3)
C21—O2—C3—C2	−178.32 (18)	N3—C9—C10—C11	179.1 (2)
C21—O2—C3—C4	1.9 (3)	C9—C10—C11—C12	−0.6 (3)
C1—C2—C3—O2	−179.31 (16)	C10—C11—C12—C13	0.2 (4)
C1—C2—C3—C4	0.5 (3)	C11—C12—C13—C14	0.3 (4)
O2—C3—C4—C5	179.65 (17)	C12—C13—C14—C9	−0.2 (4)
C2—C3—C4—C5	−0.1 (3)	C10—C9—C14—C13	−0.3 (3)
C3—C4—C5—C6	−0.5 (3)	N3—C9—C14—C13	−178.8 (2)
C4—C5—C6—C1	0.7 (3)	N1—C7—C15—C16	−89.3 (2)
C4—C5—C6—C7	−178.17 (17)	C6—C7—C15—C16	90.6 (2)
O1—C1—C6—C5	179.55 (16)	N1—C7—C15—C20	90.7 (2)
C2—C1—C6—C5	−0.3 (2)	C6—C7—C15—C20	−89.4 (2)
O1—C1—C6—C7	−1.6 (3)	C20—C15—C16—C17	0.9 (3)
C2—C1—C6—C7	178.53 (17)	C7—C15—C16—C17	−179.07 (18)
N2—N1—C7—C6	−179.56 (15)	C15—C16—C17—C18	−0.5 (3)
N2—N1—C7—C15	0.3 (3)	C16—C17—C18—C19	−0.2 (4)
C5—C6—C7—N1	177.65 (16)	C17—C18—C19—C20	0.5 (4)
C1—C6—C7—N1	−1.2 (2)	C16—C15—C20—C19	−0.6 (3)
C5—C6—C7—C15	−2.2 (3)	C7—C15—C20—C19	179.34 (19)
C1—C6—C7—C15	178.97 (15)	C18—C19—C20—C15	−0.1 (3)
C9—N3—C8—O3	8.5 (3)	C23A—N4A—C22A—O4A	5.7 (12)
C9—N3—C8—N2	−171.40 (18)	C24A—N4A—C22A—O4A	178.0 (10)
N1—N2—C8—O3	−3.9 (3)	C24B—N4B—C22B—O4B	2 (3)
N1—N2—C8—N3	175.94 (16)	C23B—N4B—C22B—O4B	−178 (2)

### Hydrogen-bond geometry (Å, º)

**Table:**

*D*—H···*A*	*D*—H	H···*A*	*D*···*A*	*D*—H···*A*
O1—H1···N1	0.89	1.78	2.5655 (18)	147
N2—H2′···O4*A*	0.87	2.19	2.941 (12)	145
N2—H2′···O4*B*	0.87	2.22	2.96 (3)	143
N3—H3′···O4*A*	0.85	2.00	2.830 (7)	165
N3—H3′···O4*B*	0.85	2.07	2.882 (18)	161
C24*A*—H24*C*···O3^i^	0.96	2.62	3.400 (4)	139
C23*B*—H23*E*···O3^i^	0.96	2.31	3.157 (13)	147
C23*B*—H23*F*···O1^i^	0.96	2.59	3.473 (13)	153

Symmetry code: (i) *x*, *y*, *z*−1.
